# The diagnosis and treatment of *Helicobacter pylori* infection in Arctic regions with a high prevalence of infection: Expert Commentary

**DOI:** 10.1017/S0950268815001181

**Published:** 2015-06-22

**Authors:** B. J. McMAHON, M. G. BRUCE, A. KOCH, K. J. GOODMAN, V. TSUKANOV, G. MULVAD, M. L. BORRESEN, F. SACCO, D. BARRETT, S. WESTBY, A. J. PARKINSON

**Affiliations:** 1 Departments of Internal Medicine and Surgery, Alaska Native Tribal Health Consortium, Anchorage, AK, USA; 2 Arctic Investigations Program, Division of Preparedness and Emerging Infections, National Center for Emerging and Zoonotic Infectious Diseases, Centers for Disease Control and Prevention, Anchorage, AK, USA; 3 Department of Epidemiology Research, Statens Serum Institute, Copenhagen, Denmark; 4 Canadian North Helicobacter pylori Working Group, University of Alberta, Edmonton, Alberta, Canada; 5 Department of State Medical Research Institute for Northern Problems, Siberian Division of Russian Academy of Medical Sciences, Krasnoyarsk, Russia; 6 Primary Health Care Clinic, Nuuk, Greenland

**Keywords:** Antibiotic resistance, health policy, *Helicobacter pylori*, gastrointestinal infections

## Abstract

*Helicobacter pylori* infection is a major cause of peptic ulcer and is also associated with chronic gastritis, mucosa-associated lymphoid tissue (MALT) lymphoma, and adenocarcinoma of the stomach. Guidelines have been developed in the United States and Europe (areas with low prevalence) for the diagnosis and management of this infection, including the recommendation to ‘test and treat’ those with dyspepsia. A group of international experts performed a targeted literature review and formulated an expert opinion for evidenced-based benefits and harms for screening and treatment of *H. pylori* in high-prevalence countries. They concluded that in Arctic countries where *H. pylori* prevalence exceeds 60%, treatment of persons with *H. pylori* infection should be limited only to instances where there is strong evidence of direct benefit in reduction of morbidity and mortality, associated peptic ulcer disease and MALT lymphoma and that the test-and-treat strategy may not be beneficial for those with dyspepsia.

## INTRODUCTION

*Helicobacter pylori* infection is a prevalent condition identified in the majority of the population in many countries around the world as well as in persons who have immigrated from high-prevalence to low-prevalence countries [1]. *H. pylori* infection is a major cause of peptic ulcer disease and is also associated with chronic gastritis, mucosa-associated lymphoid tissue (MALT) lymphoma, and adenocarcinoma of the stomach [2, 3]. In some countries, gastric cancer is one of the leading causes of cancer-related death especially in males [4].

### Prevalence

The prevalence of *H. pylori* infection ranges widely across populations, ranging from under 15% to greater than 85% of the population [5]. In certain settings infection commonly takes place in childhood and is often lifelong [1, 6]. The prevalence of *H. pylori* infection in Arctic countries is high, with very high rates (>50%) in many indigenous populations. Studies conducted in the US Arctic (Alaska) have found that the seroprevalence of *H. pylori* is significantly increased in Alaska Native (AN) Peoples (75% overall in 1983–1987 [7], and 63% 1998–2005) [8] compared to non-Native Alaskans (24%) [9] and considerably higher than *H. pylori* prevalence in persons in the contiguous 48 US states which averages between 30% and 40% [9, 10]. Similarly in the Canadian Inuit and First Nations People, prevalence up to 95% in older age groups has been reported in 2008 [11]. In Greenland, the seroprevalence was lowest in children aged 0–4 years (6%), but increased rapidly thereafter and stabilized around 58% in persons aged 15–87 years in a study conducted in 1998 [12].

### Clinical syndromes associated with *H. pylori* infection and outcomes of eradication

Eradication of *H. pylori* infection has clearly been shown to be associated with healing of pre-pyloric and duodenal ulcers and preventing their recurrence, but this is less apparent for gastric ulcers [13]. Furthermore, elimination of *H. pylori* infection has been found to result in cure of low-grade MALT lymphoma [14]. There is strong epidemiological evidence, from nested case-control studies, linking *H. pylori* infection to subsequent development of gastric cancer [4, 15]. The International Agency for Research on Cancer has declared *H. pylori* as a group 1 agent, meaning it is carcinogenic to humans [16]. The findings of randomized trials conducted to determine if eradication of *H. pylori* infection results in a decrease in the incidence of gastric carcinoma in persons in whom the infection has been eradicated compared to infected untreated controls have been conflicting [17–20]. A meta-analysis of *H. pylori* eradication and gastric cancer risk examining six randomized-controlled trials (RCTs) showed that 1·1% of treated groups *vs*. 1·7% of control groups developed gastric cancer [21]. Although reduction in relative risk was not found in any of the individual trials, when the trials were combined this meta-analysis did show overall reduction of risk. However, no cost estimate analysis was done and the number needed to treat to reduce one case of gastric cancer was not calculated. A recent report exploring the quality of RCTs of *H. pylori* eradication for the prevention of gastric cancer and pre-neoplastic lesions found that the qualities of the RCTs were questionable and the protective efficacy exaggerated in some RCTs [22]. Furthermore, recently, a well-designed prospective community-based study from Taiwan with over 4000 participants utilizing a 5-year observational period after screening, was followed by mass treatment of *those* persons with *H. pylori* followed by 5 years of further observation. The investigators found a significant reduction of the incidence of gastric atrophy (but not intestinal metaplasia) but not in the incidence of gastric cancer during the 5 years following treatment compared with the 5-year period after diagnosis prior to treatment (rate ratio 0·75, 95% confidence interval 0·372–1·524) [23].

*H. pylori* infection is associated with iron deficiency anaemia, although the degree of anaemia in the absence of peptic ulcer is usually modest [9, 10]. *H. pylori* causes differing degrees of inflammation in the gastric mucosa in persons infected with this pathogen and has also been cited as a cause of non-specific dyspepsia in humans. Primary-care providers frequently encounter persons with dyspepsia, which can result from a variety of causes. Within the differential diagnosis of dyspepsia is gastritis, secondary to *H. pylori.* However, double-blind randomized placebo-controlled trials of antimicrobial agents have either shown no benefit of eradication of *H. pylori* in persons with dyspepsia compared to infected controls in terms of subsequent reduction of symptoms [24, 25] or only a very modest benefit in a minority of participants [26, 27]. A randomized double-blind population-based study of the ‘test and treat’ strategy (*H. pylori* antibody test or breath test) demonstrated only a 5% improvement in dyspepsia symptoms [27]. The high prevalence of *H. pylori* in certain populations limits the usefulness of anti-*H. pylori* IgG antibody tests for clinical purposes, since nearly all adults will be positive. In this setting the *H. pylori* antibody test does not differentiate between an active infection in a patient with dyspeptic symptoms or a prior cleared infection as anti-*H. pylori* IgG can persist for years after an infection is over [28]. While stool antigen tests and urea breath test (UBT) can indicate active infection, in high endemic settings the causes of patients' dyspeptic symptoms may, in many instances, not be due to *H. pylori* infection but could be due to other causes such as oesophagitis or motility disorders. Finally, test-and-treat strategy also may not be beneficial in low-prevalence populations, including those living in endemic areas [29].

### Antimicrobial agents against *H. pylori,* risk of drug resistance and incidence of reinfection

Currently there are at least eight antimicrobial drugs available to treat infection with *H. pylori*: amoxicillin, tetracycline, clarithromycin, metronidazole, levofloxacin, tinidazole, furazolidone and rifabutin. Proton pump inhibitors (PPIs), histamine 2 (H2) blockers and bismuth-containing agents are adjuvant drugs that enhance the performance of antimicrobial agents. Successful eradication of infection requires the administration of two or three antimicrobial agents, simultaneously or sequentially, combined with a PPI, H2 blocker and sometimes includes a bismuth-containing compound given for 7–14 days [30, 31]. The proportion of patients in whom the infection is eradicated after treatment ranges from 60% to 95%. Worldwide, *H. pylori* antimicrobial resistance is high, and appears to be rising for at least three of these eight agents, clarithromycin, metronidazole and levofloxacin [32]. Antimicrobial resistance rates from isolates recovered from a predominately AN population were between 42% and 66% for metronidazole, 30% for clarithromycin and 8–19% for levofloxacin [33–35]. In Alaska, this may partially be due to the fact that antimicrobial agents are prescribed at a rate of three times higher than the national average [36]. The proportion of isolates from Alaska and around the world that demonstrate antimicrobial resistance to tetracycline and ampicillin remains low [36]. Little is known about resistance rates for tinidazole, furazolidone and rifabutin. In most regions, including developed regions, there are few laboratories that have the capability to conduct culture and sensitivity testing of *H. pylori* isolates; therefore information needed to select the best agents to use based on sensitivity testing is often not available. Since rates of antimicrobial resistance to agents used for treatment of dyspepsia caused by *H. pylori* are high and increasing, use of these agents in high-prevalence populations could lead to increasing antimicrobial resistance not only to *H. pylori*, but also to other common bacterial causes of infection such as *Streptococcus pneumoniae, Staphylococcus aureus*, and *Haemophilus influenzae*.

While reinfection rates are low in developed countries (under 3% per year) [37–39]; in developing countries they are high, from 10% to 50% over a 1- to 2-year period post-eradication [40–42]. A recent study in seven Latin American countries found the reinfection rate at 1 year to be 11·5% in patients who had a negative post-treatment UBT [43]. In Alaska, reinfection rates have been found to be 14·5% at 2 years post-eradication of *H. pylori* in urban AN persons, many who had emigrated previously from rural communities to the city of Anchorage [44]. Reinfection rates in persons born in high-prevalence countries who immigrated to countries with low prevalence have not been studied.

### Deficiencies of current practice guidelines

Guidelines and consensus statements for developed countries, where *H. pylori* infection is found in less than one third of the population, have been written for managing persons with *H. pylori* infection [16, 45–48]. However, while these guidelines may be appropriate for low-prevalence populations, in regions of the world where the prevalence of *H. pylori* infection is greater than 60%, the majority of persons carry *H. pylori* in their gastric mucosa, and the presence of this bacteria may not be the cause of an individual's symptoms of dyspepsia [49]. In other words, a person who does not have dyspepsia may have the same probability of harbouring *H. pylori* in his/her gastric mucosa as one who has dyspepsia. Furthermore, randomized trials from regions with low prevalence have found that treatment of *H. pylori* in persons with dyspepsia has no or little symptomatic benefit [26, 27, 50]. Guidelines for developed countries may not apply to countries and regions where the majority of people (with some estimates as high as ⩾85%) are infected [12].

Current practice guidelines advocate testing patients with dyspeptic symptoms for *H. pylori* infection primarily using non-invasive methods including serology, UBT, or stool antigen, and then treating all those who are found to test positive for this organism [16, 45, 47, 48]. It has become apparent to the experts in this Panel who work in Arctic countries/states/territories such as the US Arctic (Alaska), Northern Canada, Greenland and Arctic Russia that these guidelines, while appropriate for low-endemic populations, may not be applicable to persons living in rural settings and for Arctic Indigenous people, since the epidemiology of *H. pylori* in these settings resembles that of developing countries [5]. The test-and-treat strategy has shown only a modest benefit in relief of symptoms of dyspepsia (5%) for those living in low-endemic areas in two RCTs [26, 27], but no benefit in two others [24, 25]. Thus, it is unlikely that if the test-and-treat strategy was applied to high-prevalence countries, substantial symptomatic benefit would be achieved. Thus, in general, guidelines developed for persons living in countries where the prevalence is low may not apply to persons living in countries/regions where the prevalence of *H. pylori* infection is high.

## METHODS

Clinicians, public health experts and researchers with expertise in *H. pylori* infections in the Arctic and sub-Arctic met in Copenhagen in 2010 and 2011 and in Fairbanks, Alaska in 2012. The group consisted of three (B.J.M., M.G.B., A.J.P.) epidemiologists from the Arctic Investigations Program of the Centers for Disease Control and Prevention and four clinicians from the Alaska Native Medical Center (B.J.M., F.S., D.B., S.W.) both in Anchorage, Alaska, who had conducted and published studies on the epidemiology and outcome after treatment of *H. pylori* in Alaska, three clinicians and epidemiologists from Greenland and Denmark (A.J.K., G.M., M.L.B.), one gastroenterologist from Russia working in Siberia (V.T.) and one epidemiologist conducting studies in the Northwest Territories of Canada (K.J.G.) [5, 9, 11, 12, 34, 36, 44]. These individuals drafted an Expert commentary regarding the management of *H. pylori* infection in persons living in high-prevalence regions of the world. Members of the committee performed a Medline targeted literature review using PubMed searching for the following topics: the prevalence of *H. pylori* in endemic regions, regional patterns of antimicrobial resistance, success/failure of eradication in persons with this infection living in endemic regions *vs*. low-prevalence regions, efficacy of treatment on outcomes (peptic ulcer, dyspepsia, reduction in the incidence of gastric cancer, and reinfection rates in high-prevalence *vs*. low-prevalence regions). In drafting these comments, the members of the committee gave weight to randomized control treatment trials and longitudinal cohort studies over cross-sectional studies. This information was used along with the members' own experience and expert judgement in managing persons with *H. pylori* infection in their respective regions. Over the three face-to-face meetings, these experts agreed to only include those opinions for which there was consensus. Multiple drafts of the manuscript were circulated to all panel members; any differences were resolved by email and online discussion. The experts defined an endemic region as an area where at least 60% of the population had a positive IgG test for *H. pylori*.

This commentary regarding screening, management and treatment of *H. pylori* was developed for the benefit of primary-care providers who encounter patients with symptoms of dyspepsia who live in or were born in regions of the Arctic or other areas with a high prevalence of *H. pylori* infection.

## RESULTS

### Screening or testing for *H. pylori*

*Screening or testing for* H. pylori *infection for the routine evaluation of dyspepsia or other gastrointestinal symptoms, whether utilizing serology, UBT or other techniques, is not clinically useful or supported by clinical evidence for high prevalence populations such as in the Arctic.*

**Fig. 1.**
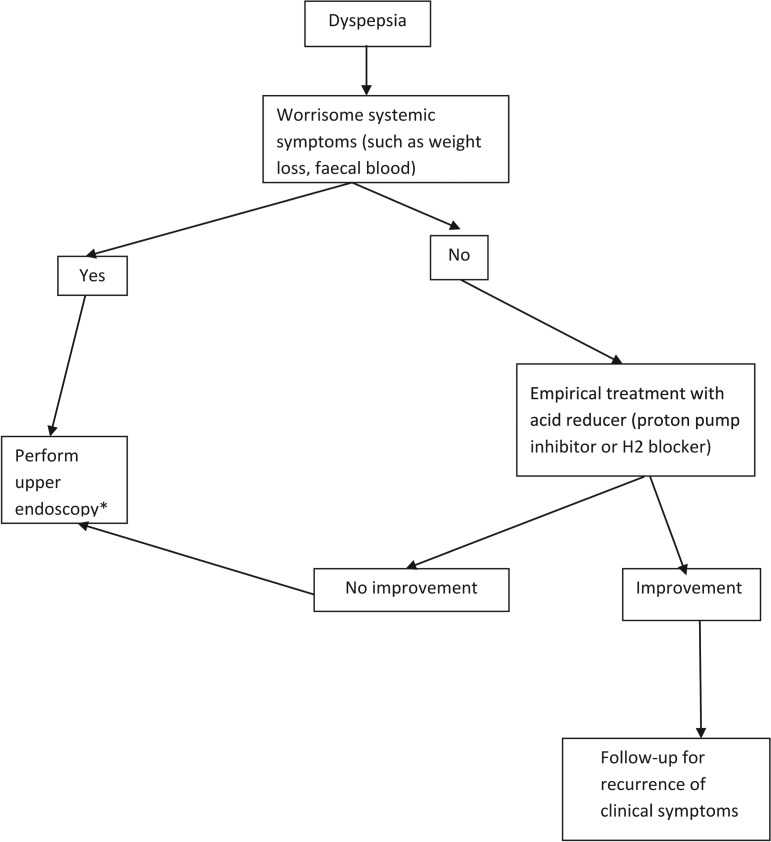
Algorithm for management of dyspepsia in regions with high prevalence (>60% population infected) of *Helicobacter pylori* infection. * Further evaluation and treatment depending on findings of pathology found on endoscopy.

*Justification.* There will be a high probability of positive serology or other test when using the test-and-treat strategy in populations with high prevalence of *H. pylori* regardless of symptomatology. In addition, controlled trials have not consistently shown that *H. pylori* eradication leads to improvement of dyspeptic symptoms [26, 27, 29]. Therefore, the test-and-treat strategy should not be utilized in populations with a high prevalence of *H. pylori.* Furthermore, the downside of treating the high percentage of persons who would be found to be positive, in regards to antimicrobial resistance could be substantial. At the same time, the Expert Panel felt that there is no evidence to justify performing endoscopy as the first step to evaluate someone with symptoms of dyspepsia (such as indigestion, bloating or heartburn) who does not have systemic symptoms or evidence of faecal blood. An algorithm for primary-care providers that can be used to help manage persons with this infection is included in Figure 1.

### Antimicrobial treatment for *H. pylori* infection

When treatment is employed, treatment should consist of regimens that have evidence of high efficacy after taking into consideration the antimicrobial resistance patterns in the region.

*Justification.* A prospective study from Alaska showed that commonly used *H. pylori* therapies recommended by authoritative clinical guidelines [16, 46, 48] did not work consistently well across populations or over time [34]. A compelling argument based on this evidence is that clinicians should use only what works locally, based on regional or locally derived antimicrobial resistance patterns [51].

### Indications for treatment of *H. pylori* infection where there is strong evidence for benefit [2, 13, 14]

(*a*) Persons with duodenal ulcers.
(*b*) Persons with gastric ulcers.
(*c*) Persons with MALT lymphoma.

*Justification.* Randomized trials and longitudinal case-series performed outside the Arctic have shown that cure of *H. pylori* results in eradication of ulcers and decreased rates of ulcer recurrence; cure of MALT lymphoma after treatment of *H. pylori* has also been well documented [2, 45, 46].

### Treatment of dyspepsia where *H. pylori* is highly prevalent

*When the goal is treatment of dyspepsia in patients from populations where* H. pylori *is highly prevalent, there is moderate evidence that* H. pylori *therapy should be undertaken only when there is endoscopic or histopathological confirmation of a condition associated with improvement following elimination of* H. pylori, *such as peptic ulcer disease.*

*Justification.* As stated above, randomized trials performed outside the Arctic have shown little or no benefit from test-and-treat strategy and evidence that eradication prevents gastric cancer is weak [26, 27, 29].

### Clinical situations where indications for treatment of *H. pylori* are inconclusive

(*a*) Severe gastritis. *For persons with severe gastritis with or without anaemia that is not associated with use of non-steroidal anti-inflammatory drugs or heavy alcohol usage, RCTs are needed to determine if treatment in these circumstances would have strong benefit.*

(*b*) Gastric cancer prevention. *The panel felt that more high-quality randomized studies with larger samples and longer terms of follow-up are needed before any recommendations regarding community-wide eradication of* H. pylori *can be made.*

*Justification (based on studies to date).* Stronger evidence from randomized trials with long-term follow-up is needed to provide information to make recommendations regarding benefits of individual and community-based programmes to reduce gastric cancer incidence and anaemia or harms such as adverse outcomes of antimicrobial resistance and cost benefits [9, 10, 22, 23].

### Clinical situations where there is not strong evidenced-based benefit for antimicrobial treatment for *H. pylori* infection but there are indications for other therapies (i.e. PPI, H2 blockers, pro-kinetic drugs)

(*a*) Persons with dyspepsia without anaemia.
(*b*) Persons with mild to moderate gastritis and oesophagitis or clear reflux symptoms.
(*c*) Persons with poor gastric motility, bezoars or conditions predisposing to gasrrointestinal motility disorder such as scleroderma or diabetes.
(*d*) *Persons with the absence of gastritis or only mild gastritis*.

*Justification.* Many causes of the symptoms of dyspepsia may not be due to *H. pylori* infection, but to other causes such as gastroesophageal reflux disease, irritable bowel or gastric motility disorders and treatment for *H. pylori* may not clinically benefit patients' symptoms [24, 25].

### Candidates for *H. pylori* treatment

*In persons who are candidates for* H. pylori *treatment, a test of cure, such as a UBT or stool antigen, 2 months or later after completion of therapy should be performed.*

*Justification.* Since studies from Alaska and elsewhere have shown that up to 10–35% of persons will fail treatment, especially if antimicrobial sensitivity testing of *H. pylori* isolates is not available, a UBT for test of cure is necessary to identify patients who need an additional course of treatment [34, 44]. Serological tests are not recommended for this purpose because antibody persistence has been demonstrated in 71% of persons 24 months after successful treatment of *H. pylori* infection [52].

## DISCUSSION

The Expert Panel also recommended some areas where more research is needed to determine if the eradication of *H. pylori* in persons and communities in endemic areas could provide clinical benefit. Studies are needed to determine if screening and treatment of *H. pylori* infection in the presence or absence of digestive disease symptoms might be justified in high-prevalence regions. This would best be done in a randomized clinical trial or public health interventions with long-term outcome analysis aimed at assessing the cost-benefit ratio of a treatment or intervention. Investigators might chose to study persons potentially at high risk for gastric cancer, such as those with a family history of gastric cancer or those who have biopsy findings of atrophic gastritis or intestinal metaplasia. Some examples might include:

(1) Studies examining if mass community screening and treatment for *H. pylori* infection in communities with a high rate of gastric cancer (or refractory iron deficiency anaemia in children) subsequently reduces the risk of these complications.
(2) Studies examining the outcome of treatment of asymptomatic individuals infected with *H. pylori*, who have a strong family history of gastric cancer (⩾2 first-degree relatives) to determine if incidence of gastric cancer can be reduced.
(3) More randomized trials to determine if treatment of *H. pylori-*infected persons with refractory iron deficiency anaemia (unresponsive to iron replacement) has benefit.
(4) Community-based studies to determine if treatment of *H. pylori-*infected persons with atrophic gastritis and/or intestinal metaplasia could reduce the incidence of gastric cancer. This could best be done under a RCT of sufficient power and length of follow-up to assess benefits. Likewise, large controlled trials with adequate follow-up to estimate the effect of treatment in reducing the risk of gastric cancer, and to identify factors that modify this effect. These studies should include adequate numbers of persons with pre-cancerous lesions including intestinal metaplasia and atrophic gastritis. Alternatively separate randomized trials could be conducted for intestinal metaplasia as well as atrophic gastritis.
(5) Prospective cohort studies to determine if treatment of *H. pylori-*infected persons with pre-cancerous lesions (e.g. gastric dysplasia, intestinal metaplasia or atrophic gastritis) will reduce the risk of the subsequent development of gastric cancer in these individuals.

Existing evidence does not clearly identify the circumstances under which treatment in endemic populations would be expected to have a reasonable cost-benefit ratio. The following research areas would be of value:

(1) Studies to identify bacterial and host factors that may better identify *H. pylori*-infected persons who would benefit from eradication. These factors might include:
  (*a*) Specific *H. pylori* genetic (e.g. pathogenicity) markers.
  (*b*) Unique molecular/biological characteristics of pre-cancerous pathology (such as atrophic gastritis and intestinal metaplasia) that might identify which factors predict a higher risk of cancer and whether eradication of *H. pylori* would decrease subsequent cancer risk.

(2) Genome-wide studies to look for host markers associated with gastric cancer in persons infected with *H. pylori.*

Application of the opinions of this Panel regarding management of *H. pylori* in individual areas of the Arctic and elsewhere will also depend on local considerations, and that researching the local costs and benefits of alternative practices will lead to improvements in the evidence base for local policies.

In conclusion, for routine clinical practice, there is insufficient evidenced-based data to support the *H. pylori* test-and-treat strategy in patients with non-specific dyspepsia or community-wide treatment to eradicate *H. pylori* for prevention of future gastric cancer. Clinicians might consider utilizing the algorithm in Figure 1 when encountering a patient with non-specific dyspepsia, especially in patients who live in or have emigrated from high-prevalence areas of the world.
